# Loop diuretics are associated with greater risk of sarcopenia in patients with non-dialysis-dependent chronic kidney disease

**DOI:** 10.1371/journal.pone.0192990

**Published:** 2018-02-15

**Authors:** Seiko Ishikawa, Shotaro Naito, Soichiro Iimori, Daiei Takahashi, Moko Zeniya, Hidehiko Sato, Naohiro Nomura, Eisei Sohara, Tomokazu Okado, Shinichi Uchida, Tatemitsu Rai

**Affiliations:** 1 Department of Nephrology, Graduate School of Medical and Dental Sciences, Tokyo Medical and Dental University, Tokyo, Japan; 2 Department of Nephrology, Tokyo Metropolitan Ohtsuka Hospital, Tokyo, Japan; 3 Department of Internal Medicine, Tohto Bunkyo Hospital, Tokyo, Japan; 4 Department of Nephrology, Nitobe Memorial Nakano General Hospital, Tokyo, Japan; The University of Tokyo, JAPAN

## Abstract

**Introduction:**

Sarcopenia, the age-related loss of muscle mass and function, frequently accompanies chronic kidney disease. The aim of this study was to clarify the prevalence and the risk factors for sarcopenia among patients with non-dialysis-dependent chronic kidney disease (NDD-CKD), focusing on the use of drugs.

**Methods:**

We conducted a cross-sectional analysis on a cohort of 260 patients with NDD-CKD in a university hospital, recruited between June 2016 and March 2017. We extracted data on patient gender, age, cause of chronic kidney disease, use of drugs, and comorbidities that could potentially affect the prevalence of sarcopenia. Sarcopenia was diagnosed using the criteria of the Asian Working Group for Sarcopenia. Logistic regression analysis was performed to analyze the association of each factor on the prevalence of sarcopenia.

**Results:**

25.0% of our study subjects had sarcopenia. Multivariable analysis revealed that an increased risk of sarcopenia was significantly associated with age, male gender, body mass index, diabetes mellitus, and loop diuretic use (odds ratio, 4.59: 95% confidence interval, 1.81–11.61: *P*-value 0.001).

**Conclusions:**

In our cohort, the prevalence of sarcopenia in patients with NDD-CKD was high, and diuretics use, particularly loop diuretic use, was suggested to be a risk factor of sarcopenia. Although loop diuretics are commonly used in patients with CKD, careful consideration of the risk of sarcopenia may be necessary.

## Introduction

Sarcopenia is defined as the age-related decline of skeletal muscle mass and function [1,2], leading to frailty, disability, hospitalization, and death [3]. Sarcopenia is a complex phenomenon with a multifactorial etiology, and the pathophysiological mechanism for its development remains unclear. The major factors considered to be involved include decreased physical activity, mitochondrial dysfunction, anorexia, loss of motor neuron end plates, and loss of anabolic hormones such as testosterone, growth hormone, and insulin-like growth factor 1, all of which tend to accompany aging. In addition, other factors including oxidative stress, insulin resistance, and an increase in proinflammatory cytokines such as interleukin-6, interleukin-1, and tumor necrosis factor alpha are also presumed to be associated with sarcopenia [2,4]. Recent studies have reported that sarcopenia is associated with a high risk of multiple adverse outcomes such as falls [5], metabolic disorders including diabetes mellitus (DM) and non-alcoholic fatty liver disease [6–8], cardiovascular events [9,10], and chronic kidney disease (CKD) [11,12]. Patients with chronic disease are likely to remain on polypharmacy (i.e., multiple drug use), which has also been reported to be associated with sarcopenia [13].

In CKD, the prevalence of sarcopenia has been reported to be 5.9% to 15.4% in non-dialysis-dependent chronic kidney disease (NDD-CKD) (CKD stage3-5) patients [12,14], and 13.7% to 33.7% in hemodialysis patients [15,16]. Multiple metabolic and nutritional abnormalities associated with increased muscle degradation and impaired muscle regeneration result in skeletal muscle loss in CKD. Several studies have shown that in addition to the general etiologic factors of sarcopenia, accumulation of uremic toxins such as indoxyl sulfate, metabolic acidosis, malnutrition, excess of angiotensin II and myostatin levels, and deficiency of vitamin D are specifically associated with sarcopenia in patients with CKD [17–19]. It has thus been suggested that patients with CKD are at risk for developing sarcopenia [3,20].

Factors such as nutritional status, inflammation, depression, and cognitive dysfunction have been reported to be associated with a higher risk of sarcopenia in CKD [16]. Likewise, polypharmacy is common among patients with CKD. Patients with CKD are frequently treated with a multi-drug regimen, usually including antihyperglycemics, antihypertensive agents, most commonly renin–angiotensin–aldosterone system (RAAS) inhibitors, antihyperuricemics, diuretics, statins, and vitamin D analogs, Although polypharmacy is considered to be a risk factor for sarcopenia, there are only limited data concerning the contribution of these drugs to the development of sarcopenia in patients with CKD, especially in patients with NDD-CKD.

In this study, we launched a cohort of patients with NDD-CKD to evaluate the prevalence of sarcopenia. The aim of the present study was to clarify the prevalence of and associated risk factors of sarcopenia using the baseline data of a cohort of patients with NDD-CKD, focusing on the use of drugs accompanying CKD treatment.

## Material and methods

### Study design and participants

This was a cross-sectional study conducted in a cohort of patients with NDD-CKD who visited the outpatient clinic at the Department of Nephrology, Tokyo Medical and Dental University Hospital. Subjects were recruited between June 2016 and March 2017. This study was approved by the ethical committee of Tokyo Medical and Dental University and was performed in accordance with the ethical principles of the Declaration of Helsinki. All subjects provided written informed consent. Inclusion of subjects in this study was based on the following criteria: (1) over 20 years of age; and (2) CKD stages 3 to 5, according to the Kidney Disease Improving Global Outcomes classification. Patients were excluded if they had (1) malignancies; (2) active infection; (3) received corticosteroid therapy before recruitment; or (4) undergone major operations no more than 6 months prior to study enrollment.

### Clinical assessments and data collection

At enrollment, subjects’ baseline clinical data, such as age, gender, height, body weight, and cause of CKD were collected. Information concerning the use of diuretics (aldosterone blockers, loop diuretics, and thiazides), DPP-4 inhibitors, RAAS inhibitors, statins, vitamin D analogs, and xanthine oxidase (XO) inhibitors, was also collected. The presence of hypertension or DM was confirmed as comorbidity. Hypertension was defined as either having systolic blood pressure ≥ 140 mmHg, diastolic blood pressure ≥ 90 mmHg, or taking antihypertensive agents. DM was defined as having hemoglobin A1c (HbA1c) level of ≥ 6.5% or taking diabetic medication. Finally, history of cardiovascular diseases (stroke, ischemic heart disease, congestive heart failure, and peripheral arterial disease), history of fractures, and exercise and smoking habits were recorded. An exercise habit was defined as a routine of one of the following exercise: aerobic exercise, resistance training, or any other sports.

### Measurements

Anthropometric measurements [triceps skinfold (TSF) and arm circumference (AC)] were measured and arm muscle circumference (AMC) was calculated as follows: AMC (cm) = AC (cm) −3.14 × TSF (cm). TSF was measured using a skinfold caliper and AC using a measuring tape. In the sitting position, keeping his/her arms straight by the sides of the body, the subject was measured for TSF and AC of the arm opposite to the dominant arm. Values were recorded as the average of three trials. Handgrip strength of the both upper limb was measured using a handheld dynamometer. In the standing position, with the arms straight by the sides, the subject gripped the instrument as hard as possible. Value (kg) was recorded as the better performance of two trials. Whether gait speed was faster than 0.8 m/s was judged by observing the subject actually walking. Blood and urine samples were collected to measure hemoglobin, albumin, blood urea nitrogen, creatinine (Cr), cystatin C (cysC), HbA1c, C-reactive protein (CRP), and urine protein-to-creatinine ratio (UPCR). estimated glomerular filtration rate (eGFR) was calculated using the modified three-variable Modification of Diet in Renal Disease equation revised by the Japanese Society of Nephrology to adjust for Japanese physical characteristics: Cr-based eGFR (eGFRcr) = 194 × serum Cr^−1.094^ × age^−0.287^ (× 0.739, if female), cysC-based eGFR (eGFRcys) = (104 × cysC^−1.019^ × 0.996^age^ (× 0.929, if female))—8. Body composition was assessed by whole-body dual-energy X-ray absorptiometry (DEXA). We quantified muscle mass using skeletal muscle mass index (SMI) calculated as follows: SMI = sum of lean mass for the arms and legs (kg) / height^2^ (m^2^).

### Diagnosis of sarcopenia

Sarcopenia was diagnosed by the criteria of the Asian Working Group for Sarcopenia (AWGS) to assess the presence of both low muscle function (low muscle strength or low physical performance) and low muscle mass. Patients meeting the following criteria were diagnosed with sarcopenia: (1) aged 65 years and older; (2) having low handgrip strength (<26 kg in males and <18 kg in females) and/or low usual gait speed (<0.8 m/s); and (3) having low SMI (<7.0 kg/m^2^ in males and <5.4 kg/m^2^ in females) [20].

### Statistical analysis

Baseline characteristics are shown as mean ± standard deviation and median with interquartile range for continuous variables; categorical variables are presented as numbers and percentages. Comparisons between the sarcopenia and the non-sarcopenia group were performed using Student’s unpaired t-test or Mann–Whitney’s *U* test for continuous variables and chi-squared test or Fisher’s exact test for categorical variables, as appropriate. Variables significantly different at *P*-values <0.10 between the sarcopenia and the non-sarcopenia group were assessed as potential confounders. To identify independent risk factors of sarcopenia, logistic regression analysis was performed. First, associations between sarcopenia and potential confounders were analyzed using univariate analysis. Then, variables associated with sarcopenia at *P*-values <0.10 in the univariate analysis were included as independent variables in multivariable analysis, and the following five statistical models were used: model adjusted for age, gender, body mass index (BMI), and eGFRcr (model 1); model adjusted for all variables in model 1 plus any type of diuretic use (model 2a) or loop diuretic use (model 2b); and model adjusted for all variables in model 2a or 2b plus DM (model 3a or 3b). Due to its skewed distribution, CRP was transformed to the natural logarithm of the actual data before the logistic regression analysis. All statistical analyses were performed using SPSS version 24 for Windows (SPSS, Inc., Chicago, IL, USA). *P*-values <0.05 were considered statistically significant.

## Results

Overall, 366 patients participated in this study. After enrollment, patients who were less than 65 years of age or lacked the measurements of handgrip strength and gait speed were excluded. Finally, 260 subjects were included in the analysis.

### Baseline characteristics and relationship between sarcopenia and various factors

**Fig 1:**
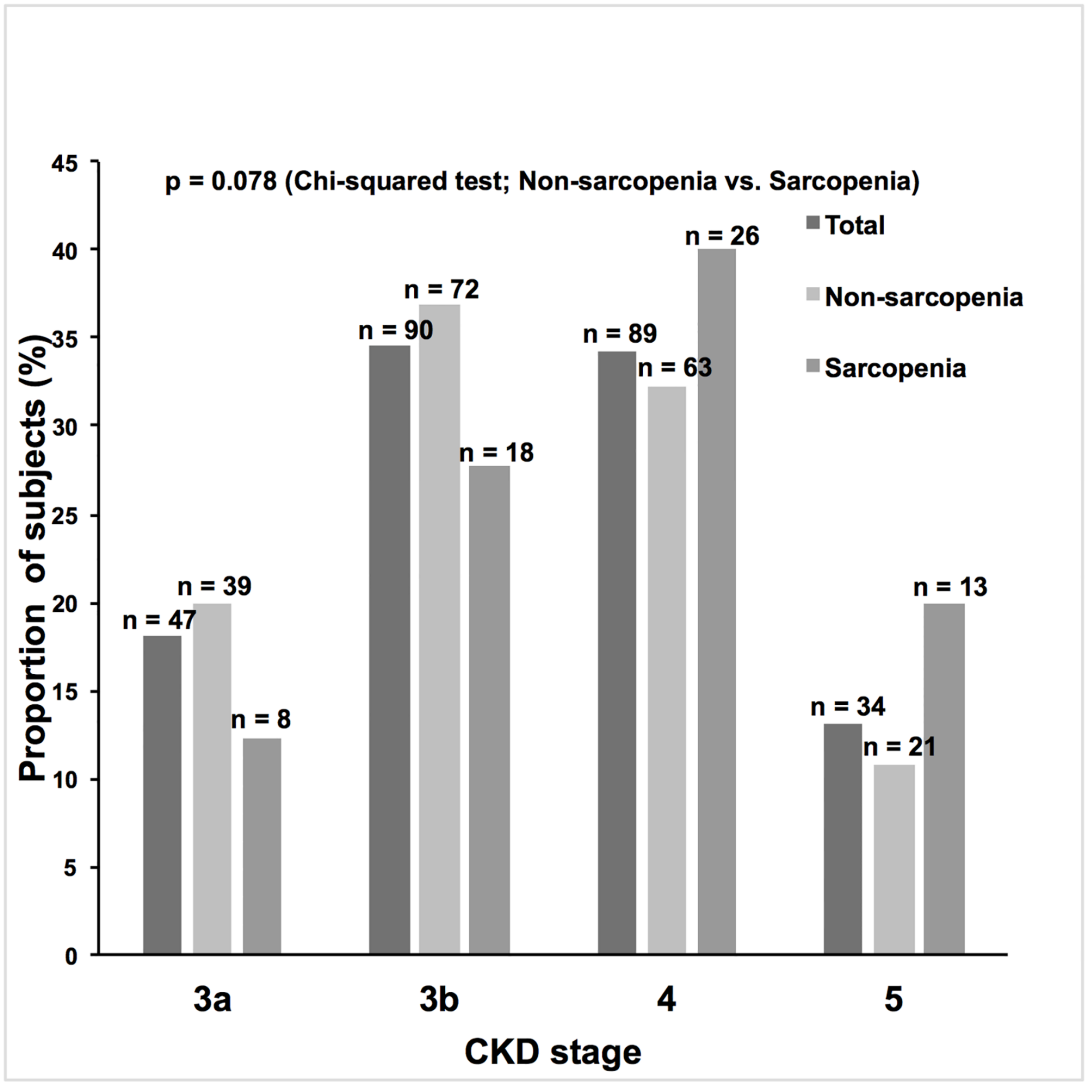
The overall proportions of subjects in each chronic kidney disease (CKD) stage (3a: eGFRcr 45–59 ml/min/1.73 m^2^; 3b: eGFRcr 30–44 ml/min/1.73 m^2^; 4: eGFRcr 15–29 ml/min/1.73 m^2^; 5: eGFRcr <15 ml/min/1.73 m^2^). The difference in proportions of subjects in each CKD stage was examined using chi-squared test (the non-sarcopenia group vs. the sarcopenia group). Although the proportion of subjects with advanced CKD stages seemed to be higher in the sarcopenia group than in the non-sarcopenia group, no statistically significant difference was observed (*P*-value 0.078).

**Fig 2:**
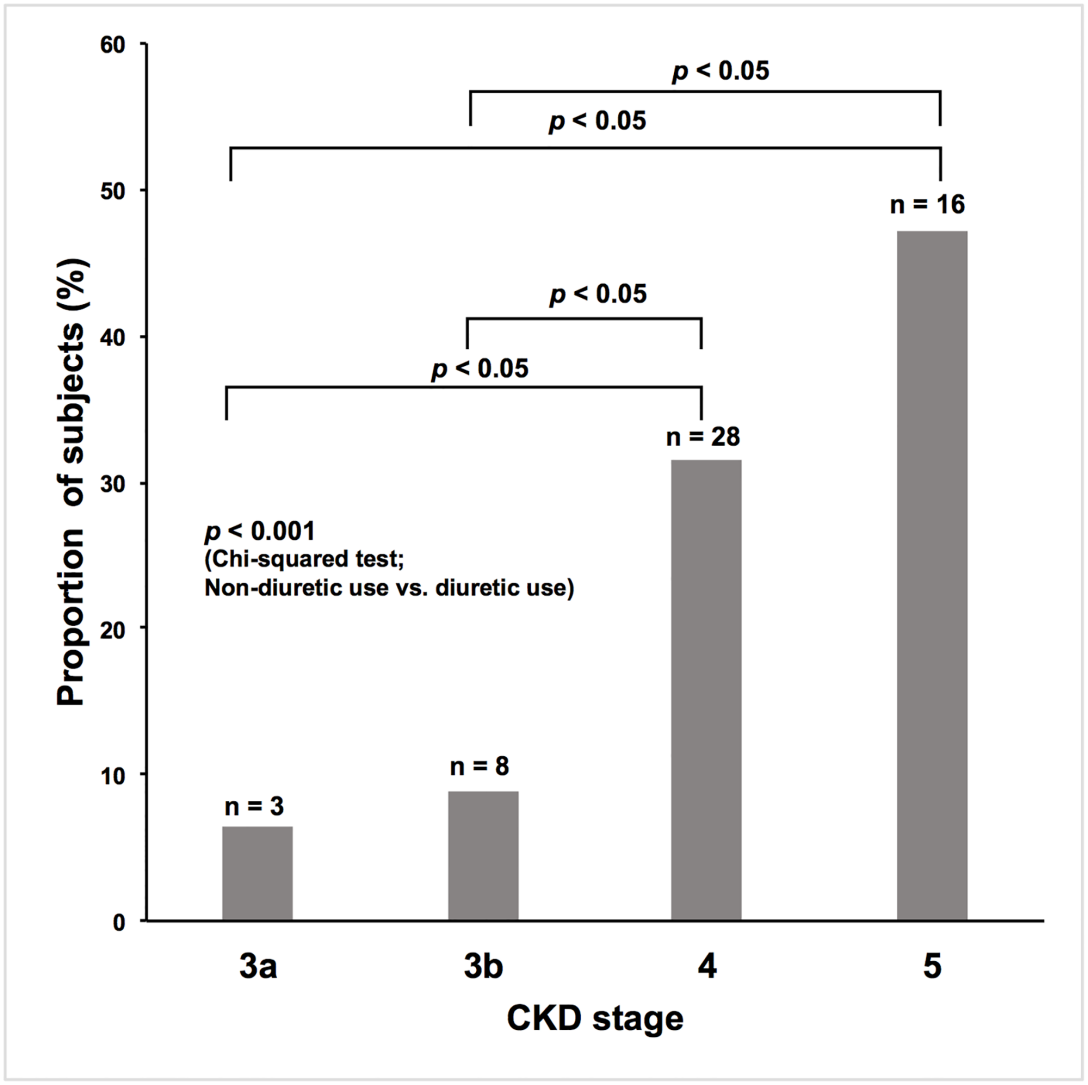
The overall proportions of subjects using any class of diuretic in each CKD stage. The differences between the proportions of subjects using any class of diuretic in each CKD stage were examined using chi-squared test (non-diuretic use vs. diuretic use) followed by a *post hoc* Bonferroni’s correction for comparisons between CKD stages. The proportions of subjects using any class of diuretic were higher in advanced CKD stages (stage 4, 5) (*P*-value <0.05).

**Fig 3:**
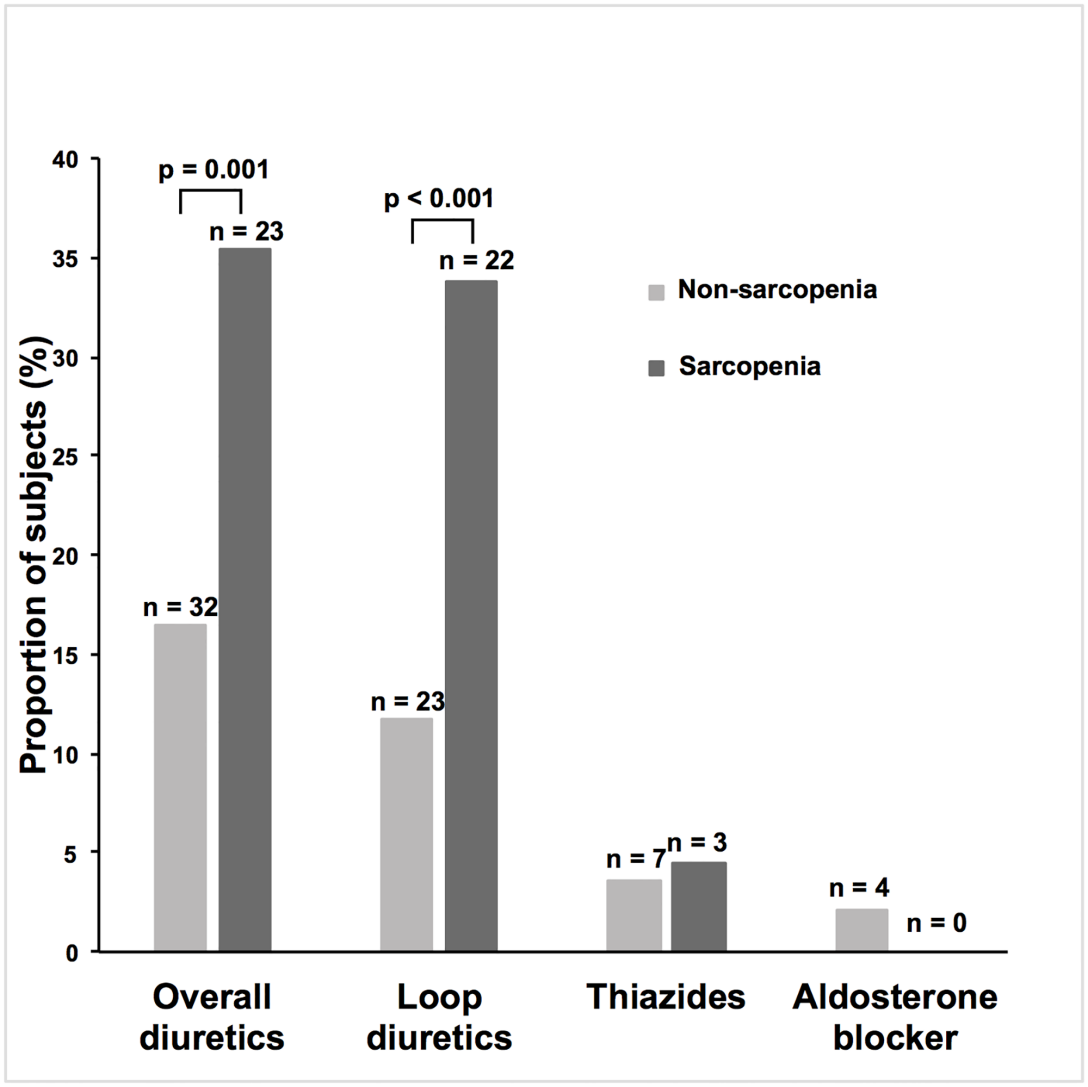
The overall proportions of subjects using each class of diuretic. The differences in proportions of subjects using each class of diuretic were examined using chi-squared test. The proportion of subjects treated with any class of diuretic was higher in the sarcopenia group (*P*-value 0.001). When different classes of diuretic were analyzed, the proportion of subjects treated with loop diuretics was higher in the sarcopenia group (*P*-value <0.001).

Clinical characteristics of the subjects are provided in Tables 1 and 2. The most common origins of CKD were benign nephrosclerosis (48.8%), chronic glomerulonephritis (19.6%), and diabetic nephropathy (13.1%). Almost half of the subjects had benign nephrosclerosis. The proportion of subjects who met the sarcopenia criteria of low handgrip strength and/or slow gait speed was 35.8%, and that of low SMI was 57.3%. When both criteria were used, 25.0% of the subjects were diagnosed with sarcopenia. Median age was higher in the sarcopenia group. The proportion of males was higher in subjects without sarcopenia (“non-sarcopenia group”), but this difference was not statistically significant. Mean eGFRcr was lower in those with sarcopenia (“sarcopenia group”). The overall proportions of subjects in each CKD stage are shown in Fig 1. Almost 50% of subjects suffered from advanced CKD stages (stages 4, 5). Although the proportion of subjects with advanced CKD stages seemed to be higher in the sarcopenia group than in the non-sarcopenia group, no statistically significant difference was observed. Median CRP level was higher in the sarcopenia group. The proportion of subjects with an exercise habit was lower in the sarcopenia group, whereas no significant difference between the two groups was observed for smoking habits. Concerning comorbidities and past history, only the prevalence of DM was significantly different between the two groups, with a higher prevalence in the sarcopenia group. The proportion of subjects using any class of diuretic was higher in the sarcopenia group, and, further, when evaluated by CKD stages, it was higher in advanced CKD stages (stage 4, 5) (Fig 2). When different classes of diuretic were analyzed, the proportion of subjects treated with loop diuretics was higher in the sarcopenia group (Fig 3). In addition, the proportion of subjects treated with XO inhibitors was significantly higher in the sarcopenia group, while no significant difference was observed in DPP-4 inhibitors, statins, or vitamin D analogs use. The proportion of subjects treated with RAAS inhibitors was marginally higher in the non-sarcopenia group (*P*-value 0.072). Mean BMI and AMC were lower in the sarcopenia group. In univariate logistic regression analysis using potential confounders, the following factors were significantly associated with the odds of having sarcopenia: age, BMI, eGFRcr, DM, overall diuretic use, loop diuretic use, XO inhibitor use, UPCR ≥ 0.5 g/gCr, hemoglobin, log CRP, and exercise habit (Table 3).

**Table 1:** Demographics and clinical characteristics of 260 elderly patients with NDD-CKD.

*Characteristic*	All	Non-sarcopenia	Sarcopenia	*P*-value
	n = 260	n = 195 (75.0%)	n = 65 (25.0%)	
Age (years)	76 (69–80)	74 (69–79)	80 (76–84)	<0.001
Male gender, n (%)	169 (65.0)	121 (62.1)	48 (73.8)	0.084
UPCR (g/gCr)	0.30 (0.14–1.12)	0.28 (0.14–0.83)	0.53 (0.16–1.83)	0.019
UPCR ≥ 0.5 (g/gCr), n (%)	106 (41.1)	71 (36.8)	35 (53.8)	0.016
Hemoglobin (g/dL)	12.6 ± 1.7	12.8 ± 1.7	12.2 ± 1.6	0.016
Serum albumin (g/dL)	4.0 ± 0.3	4.0 ± 0.3	3.9 ± 0.3	0.21
Serum creatinine (mg/dL)	1.52 (1.19–2.32)	1.47 (1.14–2.09)	1.87 (1.27–2.89)	0.009
eGFRcr (mL/min/1.73 m^2^)	31.5 ± 12.9	32.7 ± 12.6	28.1 ± 13.1	0.013
LDL cholesterol (mg/dL)	108 ± 29	108 ± 29	106 ± 28	0.57
C-reactive protein (mg/dL)	0.07 (0.04–0.18)	0.07 (0.03–0.14)	0.18 (0.05–0.35)	0.001
HbA1c (%)	6.0 (5.7–6.4)	6.0 (5.8–6.4)	6.0 (5.6–6.5)	0.95
Exercise habit, n (%)	138 (53.1)	112 (57.4)	26 (40.0)	0.015
Smoking habit, n (%)	133 (51.2)	95 (48.7)	38 (58.5)	0.17
Hypertension, n (%)	199 (76.5)	152 (77.9)	47 (72.3)	0.35
Diabetes mellitus, n (%)	72 (27.7)	46 (23.6)	26 (40.0)	0.01
History of cardiovascular disease, n (%)	71 (27.3)	49 (25.1)	22 (33.8)	0.17
History of fractures, n (%)	71 (27.3)	53 (27.2)	18 (27.7)	0.94
Overall diuretic use, n (%)	55 (21.2)	32 (16.4)	23 (35.4)	0.001
Loop diuretics, n (%)	45 (17.3)	23 (11.8)	22 (33.8)	<0.001
Overall anti-hypertensive drug use, n (%)	207 (79.6)	156 (80.0)	51 (78.5)	0.79
RAAS inhibitors, n (%)	168 (64.6)	132 (67.7)	36 (55.4)	0.072
DPP-4 inhibitor use, n (%)	36 (13.8)	25 (12.8)	11 (16.9)	0.41
Statin use, n (%)	97 (37.3)	78 (40.0)	19 (29.2)	0.12
Vitamin D analog use, n (%)	51 (19.6)	37 (19.0)	14 (21.2)	0.69
XO inhibitor use, n (%)	125 (48.1)	85 (43.6)	40 (61.5)	0.012

Data are presented as number and percentage, mean ± standard deviation, or median (interquartile range) as appropriate. CKD, chronic kidney disease; Cr, creatinine; DPP-4, dipeptidyl peptidase-4; eGFRcr, creatinine-based estimated glomerular filtration rate; HbA1c, hemoglobin A1c; LDL, low-density lipoprotein; NDD-CKD, non-dialysis-dependent chronic kidney disease; RAAS, renin–angiotensin–aldosterone system; UPCR, urine protein-to-creatinine ratio; XO, xanthine oxidase.

**Table 2:** Data of anthropometric measurements and physical function in 260 elderly patients with NDD-CKD.

*Characteristic*	All	Non-sarcopenia	Sarcopenia	*P*-value
	n = 260	n = 195 (75.0%)	n = 65 (25.0%)	
BMI (kg/m^2^)	22.8 ± 3.5	23.3 ± 3.5	21.5 ± 2.8	<0.001
SMI (kg/m^2^)	6.3 ± 1.0	6.5 ± 1.0	5.7 ± 0.8	<0.001
AMC (cm)	20.4 ± 2.7	20.7 ± 2.7	19.6 ± 2.3	0.002
Handgrip strength (kg)	26.1 ± 8.3	28.1 ± 8.2	20.1 ± 5.4	<0.001
Slow gait speed, n (%)	28 (10.8)	11 (5.7)	17 (26.2)	<0.001

Data are presented as number and percentage, mean ± standard deviation as appropriate. AMC, arm muscle circumference; BMI, body mass index; NDD-CKD, non-dialysis-dependent chronic kidney disease; SMI, skeletal muscle mass index.

**Table 3:** Unadjusted odds ratios for sarcopenia in 260 elderly patients with NDD-CKD.

	Unadjusted OR	*P*-value
	(95% CI)	
Age (per increase of 1 year)	1.13 (1.07–1.18)	<0.001
Male gender (ref = female)	1.73 (0.92–3.22)	0.086
BMI (per increase of 1 kg/m^2^)	0.85 (0.78–0.93)	<0.001
eGFRcr (per increase of 10 mL/min/1.73 m^2^)	0.75 (0.60–0.94)	0.014
Diabetes mellitus (ref = no)	2.16 (1.19–3.92)	0.011
Overall diuretic use (ref = no)	2.79 (1.4–5.26)	0.002
Loop diuretic use (ref = no)	3.83 (1.95–7.50)	<0.001
RAAS inhibitor use (ref = no)	0.59 (0.33–1.05)	0.074
XO inhibitor use (ref = no)	2.07 (1.17–3.68)	0.013
UPCR ≥ 0.5 (g/gCr) (ref = no)	2.00 (1.14–3.54)	0.017
Hemoglobin (per increase of 1 g/dL)	0.81 (0.68–0.96)	0.017
Log C-reactive protein (per increase of 1)	1.39 (1.12–1.74)	0.003
Exercise habit (ref = no)	0.49 (0.28–0.88)	0.016

BMI, body mass index; CI, confidence interval; eGFRcr, creatinine-based estimated glomerular filtration rate; NDD-CKD, non-dialysis-dependent chronic kidney disease; OR, odds ratio; RAAS, renin–angiotensin–aldosterone system; UPCR, urine protein-to-creatinine ratio; XO, xanthine oxidase.

### Risk factors independently associated with sarcopenia

The results of multivariate logistic regression analysis evaluating risk factors for sarcopenia are shown in Tables 4 and 5. Older age, male gender, and lower BMI were associated with higher prevalence of sarcopenia in all models. The adjusted odds ratio (aOR) of eGFRcr value for sarcopenia was statistically significant in model 1 but was non-significant in models 2a, 2b, 3a, and 3b. DM remained significantly associated with a higher risk of sarcopenia after adjustment for covariates. Similarly, both overall diuretic use and loop diuretic use remained associated with a higher prevalence of sarcopenia after adjustment for covariates (models 1, 2a, 2b, 3a, and 3b). Moreover, the aORs of loop diuretic use were higher than those of overall diuretic use. When CKD stage was selected as a covariate instead of eGFRcr, the results did not change (Tables 6 and 7, models 4, 5a, 5b, and 6b). Even when log CRP was added to covariates in models 1–3b to evaluate the contribution of inflammation, the same results were obtained. Log CRP was positively associated with the prevalence of sarcopenia (S1 and S2 Tables, models 7, 8a, 9a, 8b, and 9b). When eGFRcys was substituted for eGFRcr for adjustment, the aORs of both overall diuretic use and loop diuretic use remained statistically significant and the aORs of loop diuretic use were also higher than those of overall diuretic use (S3 and S4 Tables, models 10, 11a, 12a, 11b, and 12b). XO inhibitor use was marginally associated with higher prevalence of sarcopenia when adjusted for age, gender, BMI, eGFRcr, overall diuretic use, and DM (S5 Table, model 15, *P*-value 0.042). On the other hand, the aORs of RAAS inhibitor use were not statistically significant (S6 Table, models 16, 17, and 18).

**Table 4:** Adjusted odds ratios for sarcopenia in 260 elderly patients with NDD-CKD (adjusted for overall diuretic use).

	Model 1^a^		Model 2a^b^		Model 3a^c^	
	Adjusted OR	*P*-value	Adjusted OR	*P*-value	Adjusted OR	*P*-value
	(95% CI)		(95% CI)		(95% CI)	
Age (per increase of 1 year)	1.14 (1.08–1.20)	<0.001	1.13 (1.08–1.20)	<0.001	1.14 (1.08–1.21)	<0.001
Male gender (ref = female)	2.36 (1.15–4.84)	0.020	2.72 (1.29–5.75)	0.009	2.66 (1.24–5.70)	0.012
BMI (per increase of 1 kg/m^2^)	0.79 (0.71–0.89)	<0.001	0.75 (0.66–0.85)	<0.001	0.72 (0.63–0.82)	<0.001
eGFRcr (per increase of 10 mL/min/1.73 m^2^)	0.72 (0.55–0.93)	0.012	0.83 (0.63–1.10)	0.19	0.87 (0.65–1.15)	0.32
Overall diuretic use (ref = no)			3.73 (1.63–8.56)	0.002	3.08 (1.31–7.23)	0.010
Diabetes mellitus (ref = no)					2.65 (1.21–5.79)	0.014

BMI, body mass index; CI, confidence interval; eGFRcr, creatinine-based estimated glomerular filtration rate; NDD-CKD, non-dialysis-dependent chronic kidney disease; OR, odds ratio.

^a^ Model 1 adjusted for age, gender, BMI, and eGFRcr

^b^ Model 2a adjusted for all variables in model 1 plus overall diuretic use

^c^ Model 3a adjusted for all variables in model 2a plus diabetes mellitus

**Table 5:** Adjusted odds ratios for sarcopenia in 260 elderly patients with NDD-CKD (adjusted for loop diuretic use).

	Model 2b^a^		Model 3b^b^	
	Adjusted OR	*P*-value	Adjusted OR	*P*-value
	(95% CI)		(95% CI)	
Age (per increase of 1 year)	1.13 (1.07–1.20)	<0.001	1.14 (1.08–1.21)	<0.001
Male gender (ref = female)	2.58 (1.21–5.49)	0.014	2.54 (1.17–5.49)	0.018
BMI (per increase of 1 kg/m^2^)	0.75 (0.66–0.85)	<0.001	0.72 (0.63–0.82)	<0.001
eGFRcr (per increase of 10 mL/min/1.73 m^2^)	0.88 (0.66–1.16)	0.36	0.92 (0.69–1.23)	0.57
Loop diuretic use (ref = no)	5.33 (2.14–13.24)	<0.001	4.59 (1.81–11.61)	0.001
Diabetes mellitus (ref = no)			2.71 (1.23–5.98)	0.013

BMI, body mass index; CI, confidence interval; eGFRcr, creatinine-based estimated glomerular filtration rate; NDD-CKD, non-dialysis-dependent chronic kidney disease; OR, odds ratio.

^a^ Model 2b adjusted for all variables in model 1 plus loop diuretic use

^b^ Model 3b adjusted for all variables in model 2b plus diabetes mellitus

**Table 6:** Adjusted odds ratios for sarcopenia in 260 elderly patients with NDD-CKD (adjusted for CKD stage and overall diuretic use).

	Model 4^a^		Model 5a^b^		Model 6a^c^	
	Adjusted OR	*P*-value	Adjusted OR	*P*-value	Adjusted OR	*P*-value
	(95% CI)		(95% CI)		(95% CI)	
Age (per increase of 1 year)	1.14 (1.08–1.20)	<0.001	1.14 (1.08–1.20)	<0.001	1.14 (1.08–1.21)	<0.001
Male gender (ref = female)	2.33 (1.13–4.79)	0.021	2.70 (1.28–5.73)	0.009	2.62 (1.22–5.63)	0.014
BMI (per increase of 1 kg/m^2^)	0.79 (0.71–0.89)	<0.001	0.75 (0.66–0.85)	<0.001	0.72 (0.63–0.83)	<0.001
CKD stage (ref = CKD stage 5)						
CKD stage 4	0.54 (0.21–1.39)	0.21	0.62 (0.23–1.67)	0.35	0.74 (0.27–2.02)	0.56
CKD stage 3b	0.31 (0.12–0.82)	0.019	0.48 (0.17–1.37)	0.17	0.54 (0.19–1.56)	0.26
CKD stage 3a	0.29 (0.09–0.92)	0.036	0.46 (0.14–1.57)	0.22	0.58 (0.17–2.03)	0.40
Overall diuretic use (ref = no)			3.71 (1.61–8.53)	0.002	3.00 (1.27–7.07)	0.012
Diabetes mellitus (ref = no)					2.69 (1.22–5.91)	0.014

BMI, body mass index; CI, confidence interval; CKD, chronic kidney disease; eGFRcr, creatinine-based estimated glomerular filtration rate; NDD-CKD, non-dialysis-dependent chronic kidney disease; OR, odds ratio.

^a^ Model 4 adjusted for age, gender, BMI, and CKD stage

^b^ Model 5a adjusted for all variables in model 4 plus overall diuretic use

^c^ Model 6a adjusted for all variables in model 5a plus diabetes mellitus

**Table 7:** Adjusted odds ratios for sarcopenia in 260 elderly patients with NDD-CKD (adjusted for CKD stage and loop diuretic use).

	Model 5b^a^		Model 6b^b^	
	Adjusted OR	*P*-value	Adjusted OR	*P*-value
	(95% CI)		(95% CI)	
Age (per increase of 1 year)	1.14 (1.07–1.2)	<0.001	1.14 (1.08–1.21)	<0.001
Male gender (ref = female)	2.58 (1.21–5.48)	0.014	2.52 (1.17–5.46)	0.019
BMI (per increase of 1 kg/m^2^)	0.75 (0.67–0.85)	<0.001	0.72 (0.63–0.82)	<0.001
CKD stage (ref = CKD stage 5)				
CKD stage 4	0.71 (0.26–1.94)	0.51	0.85 (0.30–2.39)	0.76
CKD stage 3b	0.62 (0.21–1.84)	0.39	0.70 (0.23–2.14)	0.54
CKD stage 3a	0.53 (0.15–1.82)	0.31	0.68 (0.19–2.43)	0.55
Loop diuretic use (ref = no)	5.29 (2.12–13.21)	<0.001	4.44 (1.74–11.34)	0.002
Diabetes mellitus (ref = no)			2.72 (1.23–6.03)	0.014

BMI, body mass index; CI, confidence interval; CKD, chronic kidney disease; eGFRcr, creatinine-based estimated glomerular filtration rate; NDD-CKD, non-dialysis-dependent chronic kidney disease; OR, odds ratio.

^a^ Model 5b adjusted for all variables in model 4 plus loop diuretic use

^b^ Model 6b adjusted for all variables in model 5b plus diabetes mellitus

## Discussion

In the present cross-sectional study, we investigated the prevalence of and associated risk factors for sarcopenia in patients with NDD-CKD, especially focusing on the relationship between sarcopenia and drugs frequently administered to patients with CKD. We found that approximately one-quarter of this cohort of patients with NDD-CKD met the definitions of sarcopenia based on the criteria of AWGS, which consist of low handgrip strength, slow gait speed, and low SMI. In multivariable logistic regression model, age, gender, BMI, eGFR, DM, CRP, and overall diuretic use (particularly loop diuretic use) were significantly associated with sarcopenia. XO inhibitor use was marginally associated with sarcopenia.

The prevalence of sarcopenia in our study was 25.0%. To our knowledge, only two other published studies have investigated sarcopenia in a cohort of patients with NDD-CKD (CKD stages 3–5) using skeletal muscle mass for the diagnosis of sarcopenia. Pereira et al. diagnosed sarcopenia with both skeletal muscle mass measured using a bioimpedance analyzer and handgrip strength, and reported its prevalence to be 5.9% [12]. Moon et al. diagnosed sarcopenia only with skeletal muscle mass measured using DEXA, and reported its prevalence to be 15.4% [14]. The latter study might have over-diagnosed sarcopenia because handgrip strength was not included in the diagnostic criteria. Nevertheless, the prevalence of sarcopenia in the two studies were lower than that of our study. The fact that our study subjects tended to be older than those of the aforementioned studies likely accounts for this discrepancy. Median age was 76 (interquartile range, 69–80) years in our study, compared to approximately 60 years in the other studies [12,14].

In our study, risk factors significantly associated with an increased risk of sarcopenia in patients with CKD were DM, diuretic (particularly loop diuretic) use, and XO inhibitor use. The association between DM and sarcopenia remained significant after adjustment for covariates including either overall diuretic use or loop diuretic use. It is well known that DM is a risk factor for sarcopenia [6,21] and our results are consistent with those of previous results.

Concerning the relationship between the drugs commonly used in patients with CKD, our study demonstrated that both overall diuretic use and loop diuretic use were associated with higher risk of sarcopenia even after adjustment for potential confounders. The association of the loop diuretic use was stronger. Mandai et al. have reported that loop diuretics suppressed skeletal muscle differentiation by blocking Na^+^-K^+^-2Cl^-^ cotransporter 1 (NKCC1), which was highly expressed in skeletal muscle by using murine skeletal muscle cells *in vitro* and *in vivo*. Thus, NKCC1 was presumed to play an essential role in myogenesis [22], and it is possible that use of loop diuretics in patients with CKD may elevate the risk of sarcopenia. To date, our study is the first to our knowledge to demonstrate the effect of loop diuretics on risk of developing sarcopenia in a population-based sample.

Regarding XO inhibitors, several previous studies have shown that XO is involved in the loss of muscle mass through oxidative stress, and XO inhibitor use prevents muscle atrophy in rats [23,24]. It has also been also reported that XO inhibitor use improved muscle function in a retrospective cohort of older rehabilitation patients [25]. In our study XO inhibitor use was rather associated with a rather higher risk of sarcopenia with marginal significance, However, this was only demonstrated in a model adjusted for covariates including DM (S5 Table, model 15) and the magnitude of association was quite small. Obviously, further studies are required to assess the relationship between XO inhibitor use and sarcopenia in CKD.

Previous studies have demonstrated that use of RAAS inhibitors was associated with a lower risk of sarcopenia [26,27]. In our study, use of RAAS inhibitors showed marginal significance for a decreased risk of sarcopenia in univariate logistic regression analysis (Table 3). However, this association was not apparent after adjustment for confounders (S6 Table, models 16, 17, and 18).

We also sought to identify associations of use of DPP-4 inhibitors, statins, or vitamin D analogs with sarcopenia. DPP-4 inhibitors have shown beneficial effects on skeletal muscle [28]; on the other hand, statins have been suggested to have muscular toxicity and induce muscle weakness [29,30]. Supplementation with vitamin D analogs has been suggested to have a positive effect on skeletal muscle dysfunction in populations with CKD; however, those studies describing efficacy of vitamin D analogs had small sample sizes [31–33]. In our study, no significant difference was observed in the proportions of subjects treated with any of these three agents between the sarcopenia and the non-sarcopenia group (Table 1). The relatively low sample size of this study may have affected the results of statistical analysis. Further studies are needed to establish the relationship of specific drug use and sarcopenia in CKD patients.

We also observed that in multivariate analysis, the association between renal function and sarcopenia dissipated after adjustment for either overall diuretic use or loop diuretic use (Tables 4–6). This reflects the effects of both overall diuretic use and loop diuretic use on renal function as confounders, since many of our subjects tended to have advanced renal dysfunction and needed to be treated with some class of diuretic for volume control as shown in Fig 2. The possible association between renal function and sarcopenia is controversial; several studies have reported that decreased renal function was a risk factor for sarcopenia [14,34–36], whereas others did not find such an association, coinciding with the findings of our study [9,37]. We assume that one of the reasons for this discrepancy is due to the difference in the proportion of patients with advanced renal dysfunction among the studies.

In Tables 1–7, we evaluated renal function using eGFRcr. Since serum Cr levels may be affected by muscle mass [38], we considered the possibility that renal function may have been overestimated in the sarcopenia group. Recently, cysC has become more prevalent as a biomarker of renal function because compared to Cr, its concentration is less strongly influenced by muscle mass [39]. Therefore, we analyzed the data with eGFRcys as well. Results confirmed that after adjustment for potential confounders, eGFRcys was not associated with sarcopenia, whereas loop diuretic use was strongly associated with sarcopenia (S3 and S4 Tables). Nevertheless, we deemed eGFRcys to be inadequate to correctly evaluate renal function in our sample. This judgment was made because it is known that unlike serum Cr, serum cysC does not increase in association with a reduction of GFR in patients with advanced CKD [40], whereas in our study almost half of subjects belonged to CKD stages 4 and 5. Although there are difficulties in the accurate evaluation of renal function of patients with CKD with sarcopenia, the main results of our study were consistent regardless of which parameter, eGFRcr or eGFRcys, was used for evaluation of renal function.

Our study had several limitations. First, because of the cross-sectional nature of the study, we could not reveal causal relationships between sarcopenia and diuretic use. We also could not consider effects of the duration of diuretic use on the risk of sarcopenia. Further prospective studies will be necessary to elucidate these points. Second, the current study was conducted in a university hospital in Tokyo, and results should not be generalized to subjects of other races or nationalities. Third, the levels of inflammatory cytokines other than CRP were not measured in this study; thus, we could not thoroughly evaluate the association between inflammation status and sarcopenia. Finally, we had no information on the fluid volume status, and cardiac function of subjects. We, therefore, could not exclude the possibility that hypervolemic status or reduced cardiac function, which commonly accompany diuretic use, may have partly contributed to our results.

In conclusion, loop diuretic use was associated with increased risk of sarcopenia in patients with NDD-CKD, whereas DPP-4 inhibitor, RAAS inhibitor, statin, and vitamin D analog use was not. The relationship between sarcopenia and XO inhibitor use was controversial and further prospective studies are necessary to clarify this. Moreover, eGFRcr was not associated with risk of sarcopenia after adjustment for potential confounders, including loop diuretic use, and the same results were obtained when eGFRcys or CKD stage was used instead of eGFRcr as a parameter of renal function. Our data showed that DM was also associated with sarcopenia, similar to previous reports [6,21]. To our knowledge, this is the first study to show the risks of sarcopenia associated with use of loop diuretics in a cohort of patients with NDD-CKD. Since loop diuretics are commonly used in patients with advanced CKD for the treatment of volume overload, careful consideration of the risk of sarcopenia may be necessary in such patients.

## Supporting information

S1 Table Adjusted odds ratios for sarcopenia in 260 elderly patients with NDD-CKD (adjusted for overall diuretic use). (PDF)

S2 Table Adjusted odds ratios for sarcopenia in 260 elderly patients with NDD-CKD (adjusted for loop diuretic use). (PDF)

S3 Table Adjusted odds ratios for sarcopenia in 260 elderly patients with NDD-CKD (adjusted for cystatin C-based eGFR and overall diuretic use). (PDF)

S4 Table Adjusted odds ratios for sarcopenia in 260 elderly patients with NDD-CKD (adjusted for cystatin C-based eGFR and loop diuretic use). (PDF)

S5 Table Adjusted odds ratios for sarcopenia in 260 elderly patients with NDD-CKD (adjusted for overall diuretic use). (PDF)

S6 Table Adjusted odds ratios for sarcopenia in 260 elderly patients with NDD-CKD (adjusted for overall diuretic use). (PDF)
