# Supplementary material for: Loop diuretics are associated with greater risk of sarcopenia in patients with non-dialysis-dependent chronic kidney disease
Source: PLoS One. 2018 Feb 15;13(2):e0192990. doi: 10.1371/journal.pone.0192990 (PMC5814019; doi:10.1371/journal.pone.0192990)
Supplement: S1 Table — (PDF) [file pone.0192990.s001.pdf]

S1 Table. Adjusted odds ratios for sarcopenia in 260 elderly patients with NDD-CKD (adjusted for overall diuretic use)

	**Model 7**[a]		**Model 8a**[b]		**Model 9a**[c]	
	Adjusted OR (95% CI)	*P*-value	Adjusted OR (95% CI)	*P*-value	Adjusted OR (95% CI)	*P*-value
Age (per increase of 1 year)	1.13 (1.07–1.19)	<0.001	1.13 (1.07–1.19)	<0.001	1.13 (1.07–1.20)	<0.001
Male gender (ref = female)	2.26 (1.09–4.70)	0.029	2.71 (1.26–5.83)	0.011	2.64 (1.21–5.77)	0.015
BMI (per increase of 1 kg/m^2)	0.78 (0.69–0.88)	<0.001	0.74 (0.65–0.84)	<0.001	0.71 (0.62–0.81)	<0.001
eGFRcr (per increase of 10 mL/min/1.73 m^2)	0.74 (0.57–0.96)	0.024	0.85 (0.64–1.13)	0.28	0.89 (0.67–1.19)	0.44
Log C-reactive protein (per increase of 1)	1.34 (1.05–1.72)	0.020	1.36 (1.06–1.76)	0.017	1.34 (1.03–1.73)	0.028
Overall diuretic use (ref = no)			3.88 (1.66–9.05)	0.002	3.24 (1.35–7.74)	0.008
Diabetes mellitus (ref = no)					2.50 (1.13–5.51)	0.023

BMI, body mass index; CI, confidence interval; eGFRcr, creatinine-based estimated glomerular filtration rate; NDD-CKD, non-dialysis-dependent chronic kidney disease; OR, odds ratio.

[a] Model 7 adjusted for age, gender, BMI, eGFRcr, and log C-reactive protein

[b] Model 8a adjusted for all variables in model 7 plus overall diuretic use

[c] Model 9a adjusted for all variables in model 8a plus diabetes mellitus
