# Supplementary material for: Loop diuretics are associated with greater risk of sarcopenia in patients with non-dialysis-dependent chronic kidney disease
Source: PLoS One. 2018 Feb 15;13(2):e0192990. doi: 10.1371/journal.pone.0192990 (PMC5814019; doi:10.1371/journal.pone.0192990)
Supplement: S2 Table — (PDF) [file pone.0192990.s002.pdf]

S2 Table. Adjusted odds ratios for sarcopenia in 260 elderly patients with NDD-CKD (adjusted for loop diuretic use)

	Model 8b[a]		Model 9b[b]	
	Adjusted OR (95% CI)	*P*-value	Adjusted OR (95% CI)	*P*-value
Age (per increase of 1 year)	1.13 (1.07–1.19)	<0.001	1.14 (1.07–1.20)	<0.001
Male gender (ref = female)	2.55 (1.18–5.51)	0.018	2.50 (1.14–5.51)	0.022
BMI (per increase of 1 kg/m^2)	0.73 (0.65–0.84)	<0.001	0.71 (0.61–0.81)	<0.001
eGFRcr (per increase of 10 mL/min/1.73 m^2)	0.91 (0.68–1.21)	0.51	0.95 (0.71–1.28)	0.74
Log C-reactive protein (per increase of 1)	1.36 (1.05–1.76)	0.018	1.34 (1.03–1.74)	0.029
Loop diuretic use (ref = no)	5.57 (2.19–14.19)	<0.001	4.83 (1.86–12.55)	0.001
Diabetes mellitus (ref = no)			2.57 (1.15–5.71)	0.021

BMI, body mass index; CI, confidence interval; eGFRcr, creatinine-based estimated glomerular filtration rate; NDD-CKD, non-dialysis-dependent chronic kidney disease; OR, odds ratio.

[a] Model 8b adjusted for all variables in model 7 plus loop diuretic use

[b] Model 9b adjusted for all variables in model 8b plus diabetes mellitus
