# Supplementary material for: Loop diuretics are associated with greater risk of sarcopenia in patients with non-dialysis-dependent chronic kidney disease
Source: PLoS One. 2018 Feb 15;13(2):e0192990. doi: 10.1371/journal.pone.0192990 (PMC5814019; doi:10.1371/journal.pone.0192990)
Supplement: S3 Table — (PDF) [file pone.0192990.s003.pdf]

S3 Table. Adjusted odds ratios for sarcopenia in 260 elderly patients with NDD-CKD (adjusted for cystatin C-based eGFR and overall diuretic use)

	**Model 10**[a]		**Model 11a**[b]		**Model 12a**[c]	
	Adjusted OR (95% CI)	*P*-value	Adjusted OR (95% CI)	*P*-value	Adjusted OR (95% CI)	*P*-value
Age (per increase of 1 year)	1.13 (1.07–1.19)	<0.001	1.13 (1.07–1.19)	<0.001	1.13 (1.07–1.20)	<0.001
Male gender (ref = female)	2.18 (1.04–4.58)	0.040	2.55 (1.18–5.54)	0.018	2.51 (1.14–5.52)	0.022
BMI (per increase of 1 kg/m^2)	0.78 (0.69–0.88)	<0.001	0.74 (0.65–0.84)	<0.001	0.72 (0.62–0.82)	<0.001
eGFRcys (per increase of 10 mL/min/1.73 m^2)	0.67 (0.53–0.85)	0.001	0.76 (0.59–0.97)	0.029	0.78 (0.61–1.01)	0.060
Log C-reactive protein (per increase of 1)	1.28 (0.99–1.65)	0.058	1.31 (1.01–1.69)	0.043	1.29 (0.99–1.67)	0.060
Overall diuretic use (ref = no)			3.09 (1.30–7.34)	0.011	2.64 (1.09–6.40)	0.032
Diabetes mellitus (ref = no)					2.33 (1.05–5.15)	0.037

BMI, body mass index; CI, confidence interval; eGFRcys, cystatin C-based estimated glomerular filtration rate; NDD-CKD, non-dialysis-dependent chronic kidney disease; OR, odds ratio.

[a] Model 10 adjusted for age, gender, BMI, eGFRcys, and log C-reactive protein

[b] Model 11a adjusted for all variables in model 10 plus overall diuretic use

[c] Model 12a adjusted for all variables in model 11a plus diabetes mellitus
