# Supplementary material for: Loop diuretics are associated with greater risk of sarcopenia in patients with non-dialysis-dependent chronic kidney disease
Source: PLoS One. 2018 Feb 15;13(2):e0192990. doi: 10.1371/journal.pone.0192990 (PMC5814019; doi:10.1371/journal.pone.0192990)
Supplement: S4 Table — (PDF) [file pone.0192990.s004.pdf]

S4 Table. Adjusted odds ratios for sarcopenia in 260 elderly patients with NDD-CKD (adjusted for cystatin C-based eGFR and loop diuretic use)

	**Model 11b**[a]		**Model 12b**[b]	
	Adjusted OR (95% CI)	*P*-value	Adjusted OR (95% CI)	*P*-value
Age (per increase of 1 year)	1.13 (1.07–1.19)	<0.001	1.13 (1.07–1.20)	<0.001
Male gender (ref = female)	2.44 (1.12–5.31)	0.024	2.42 (1.10–5.34)	0.028
BMI (per increase of 1 kg/m^2)	0.74 (0.65–0.84)	<0.001	0.71 (0.62–0.82)	<0.001
eGFRcys (per increase of 10 mL/min/1.73 m^2)	0.79 (0.62–1.02)	0.067	0.82 (0.64–1.06)	0.13
Log C-reactive protein (per increase of 1)	1.31 (1.01–1.70)	0.043	1.29 (0.99–1.68)	0.060
Loop diuretic use (ref = no)	4.29 (1.67–11.04)	0.003	3.79 (1.45–9.94)	0.007
Diabetes mellitus (ref = no)			2.36 (1.06–5.27)	0.035

BMI, body mass index; CI, confidence interval; eGFRcys, cystatin C-based estimated glomerular filtration rate; NDD-CKD, non-dialysis-dependent chronic kidney disease; OR, odds ratio.

[a] Model 11b adjusted for all variables in model 10 plus loop diuretic use

[b] Model 12b adjusted for all variables in model 11b plus diabetes mellitus
