# Supplementary material for: Loop diuretics are associated with greater risk of sarcopenia in patients with non-dialysis-dependent chronic kidney disease
Source: PLoS One. 2018 Feb 15;13(2):e0192990. doi: 10.1371/journal.pone.0192990 (PMC5814019; doi:10.1371/journal.pone.0192990)
Supplement: S5 Table — (PDF) [file pone.0192990.s005.pdf]

S5 Table. Adjusted odds ratios for sarcopenia in 260 elderly patients with NDD-CKD (adjusted for overall diuretic use)

	Model 13[a]		Model 14[b]		Model 15[c]	
	Adjusted OR (95% CI)	*P*-value	Adjusted OR (95% CI)	*P*-value	Adjusted OR (95% CI)	*P*-value
Age (per increase of 1 year)	1.14 (1.08-1.20)	<0.001	1.14 (1.08-1.20)	<0.001	1.14 (1.08-1.21)	<0.001
Male gender (ref = female)	2.26 (1.09-4.67)	0.028	2.63 (1.23-5.61)	0.012	2.57 (1.19-5.59)	0.017
BMI (per increase of 1 kg/m^2)	0.78 (0.69-0.88)	<0.001	0.74 (0.65-0.84)	<0.001	0.70 (0.61-0.81)	<0.001
eGFRcr (per increase of 10 mL/min/1.73 m^2)	0.78 (0.59-1.03)	0.083	0.91 (0.68-1.23)	0.55	0.97 (0.72-1.32)	0.86
XO inhibitor use (ref = no)	1.87 (0.93-3.74)	0.078	1.92 (0.94-3.92)	0.075	2.15 (1.03-4.48)	0.042
Overall diuretic use (ref = no)			3.80 (1.64-8.82)	0.002	3.13 (1.32-7.45)	0.010
Diabetes mellitus (ref = no)					2.93 (1.32-6.54)	0.008

BMI, body mass index; CI, confidence interval; eGFRcr, creatinine-based estimated glomerular filtration rate; NDD-CKD, non-dialysis-dependent chronic kidney disease; OR, odds ratio; XO, xanthine oxidase.

[a] Model 13 adjusted for age, gender, BMI, eGFRcr, and XO inhibitor use

[b] Model 14 adjusted for all variables in model 13 plus overall diuretic use

[c] Model 15 adjusted for all variables in model 14 plus diabetes mellitus
