# Supplementary material for: Loop diuretics are associated with greater risk of sarcopenia in patients with non-dialysis-dependent chronic kidney disease
Source: PLoS One. 2018 Feb 15;13(2):e0192990. doi: 10.1371/journal.pone.0192990 (PMC5814019; doi:10.1371/journal.pone.0192990)
Supplement: S6 Table — (PDF) [file pone.0192990.s006.pdf]

S6 Table. Adjusted odds ratios for sarcopenia in 260 elderly patients with NDD-CKD (adjusted for overall diuretic use)

	**Model 16[a]**		**Model 17[b]**		**Model 18[c]**	
	Adjusted OR (95% CI)	*P*-value	Adjusted OR (95% CI)	*P*-value	Adjusted OR (95% CI)	*P*-value
Age (per increase of 1 year)	1.14 (1.08–1.20)	<0.001	1.13 (1.07–1.20)	<0.001	1.14 (1.08–1.20)	<0.001
Male gender (ref = female)	2.36 (1.15–4.86)	0.020	2.74 (1.29–5.83)	0.009	2.65 (1.23–5.71)	0.013
BMI (per increase of 1 kg/m^2)	0.80 (0.71–0.89)	<0.001	0.76 (0.67–0.86)	<0.001	0.73 (0.63–0.83)	<0.001
eGFRcr (per increase of 10 mL/min/1.73 m^2)	0.71 (0.54–0.92)	0.010	0.82 (0.62–1.08)	0.16	0.86 (0.64–1.14)	0.29
RAAS inhibitor use (ref = no)	0.73 (0.38–1.40)	0.34	0.75 (0.38–1.47)	0.40	0.72 (0.36–1.43)	0.35
Overall diuretic use (ref = no)			3.68 (1.61–8.44)	0.002	3.03 (1.29–7.09)	0.011
Diabetes mellitus (ref = no)					2.70 (1.23–5.93)	0.013

BMI, body mass index; CI, confidence interval; eGFRcr, creatinine-based estimated glomerular filtration rate; NDD-CKD, non-dialysis-dependent chronic kidney disease; OR, odds ratio; RAAS, renin–angiotensin–aldosterone system.

[a] Model 16 adjusted for age, gender, BMI, eGFRcr, and RAAS inhibitor use

[b] Model 17 adjusted for all variables in model 16 plus overall diuretic use

[c] Model 18 adjusted for all variables in model 17 plus diabetes mellitus
